# Putative chemical cue from *Gyrodactylus-*infected guppies subtly alters behaviour but prior exposure decreases parasite intensity

**DOI:** 10.1017/S0031182023000136

**Published:** 2023-04

**Authors:** Katrina Di Bacco, Marilyn E. Scott

**Affiliations:** 1 Institute of Parasitology, McGill University, 21111 Lakeshore Rd, Ste-Anne-de-Bellevue, QC, Canada; 2 Faculty of Veterinary Medicine, University of Montreal, 3200 Rue Sicotte, Saint-Hyacinthe, QC, Canada

**Keywords:** Behavioural modification, guppy–*Gyrodactylus* model, outbreak intensity, putative infection cues

## Abstract

The reliance on chemical communication is well established for evading predation in aquatic systems. Only a few studies have found evidence that chemical cues released from aquatic animals infected with parasites alter behaviour. Furthermore, the link between putative chemical cues and susceptibility to infection has not been studied. The objectives of this study were to determine if exposure to chemical cues from *Gyrodactylus turnbulli*-infected guppies (*Poecilia reticulata*) at various times post-infection resulted in altered behaviour of uninfected conspecifics, and if prior exposure to this putative infection cue reduced transmission. Guppies responded to this chemical cue. Those exposed for 10 min to cues released from fish that had been infected for 8 or 16 days spent less time in the centre half of the tank. Continuous exposure to infection cues for 16 days did not alter guppy shoal behaviour but provided partial protection against infection when the parasite was introduced. Shoals exposed to these putative infection cues became infected, but infection intensity increased more slowly and to a lower peak compared with shoals exposed to the control cue. These results indicate that guppies show subtle behavioural responses to infection cues, and that exposure to infection cues reduces the intensity of outbreaks.

## Graphical abstract

**Figure:**
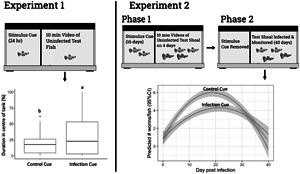

## Introduction

Animals must carefully balance how much energy is used for daily survival while protecting themselves from predators, parasites and pathogens (Lind and Cresswell, 2005). In aquatic environments, dependence on vision is unreliable as water turbidity, sedimentation and changing levels of light impact visibility (Hickman *et al*., 2004). Similarly, dependence on chemical cues can be unreliable as they are disturbed by certain water conditions such as water flow and pH (Kleinhappel *et al*., 2019; Reynolds *et al*., 2019). However, chemical cues are uninterrupted by poor water clarity and can travel further, allowing aquatic organisms to forage, find spawning grounds, mate and evade predation (Chivers *et al*., 2012). Thus, aquatic animals have evolved to use a combination of visual cues and chemical cues to understand their environment (Stauffer and Semlitsch, 1993).

A well-studied example is predator alarm cues (Brown, 2003), which are released from punctured epithelial cells when an aquatic organism is bitten by a predator (Mathuru, 2016). Alarm cues are recognized by conspecifics and induce anti-predator behaviours, such as hiding at the bottom of the substrate, darting back and forth, freezing and shoaling (Mathuru, 2016; Wisenden, 2019).

Evidence is now emerging that chemical cues released from infected individuals can also alter the behaviour of uninfected conspecifics. When juvenile rainbow trout (*Oncorhynchus mykiss*) were exposed to conspecifics during active infection with cercariae of the trematode *Diplostomum* sp., their darting and freezing behaviours increased (Poulin *et al*., 1999) suggesting that the damage caused by cercarial penetration resulted in the release of chemical cues from the fish epithelium. When bullfrog (*Rana catesbeiana*) tadpoles were exposed to either chemical or visual cues of conspecifics infected with the fungus, *Candida humicola*, the tadpoles responded only to the chemical cues and shifted their position away from the infection, suggesting that chemical cues were more reliable than visual cues (Kiesecker *et al*., 1999). Similarly, when guppies (*Poecilia reticulata*) were exposed to chemical or visual cues of a conspecific infected with the monogenean, *Gyrodactylus turnbulli*, they avoided the chemical but not visual cues, but only at 16 days after infection when transmission risk was highest. This chemical cue seemed to be host derived as it was the duration of the infection, and not the parasite load which elicited a response (Stephenson *et al*., 2018). Additionally, it has been demonstrated that guppies can imprint on chemical infection cues, resulting in a maladaptive preference for spending more time with infected conspecifics (Stephenson and Reynolds, 2016). Together, these studies suggest that infection cues may not only influence behaviour but may also impact epidemic dynamics of fish responding to these putative cues. Exploring this phenomenon in the guppy–*Gyrodactylus* model system is particularly interesting given that *G. turnbulli* live on the surface of the skin (Harris, 1986) and may release a host-mediated chemical cue over a prolonged period, contrasting temporary cues released during predation or cercarial penetration when skin cells are ruptured (Rohr *et al*., 2009).

### Study system

The guppy (*P. reticulata*) is a small tropical fish native to rivers, streams and ponds in Trinidad where it has been used as a model for evolutionary biology (Gosline and Rodd, 2008; Gotanda *et al*., 2013). The ectoparasite *G. turnbulli* infects wild guppies and is a well-known pathogen of aquarium guppies (King and Cable, 2007). This small (~800 *μ*m × 300 *μ*m) parasite (Harris, 1986) ingests epithelial and mucus cells (Bakke *et al*., 2007). It is directly transmitted between guppies during physical contact (Bakke *et al*., 2007). *Gyrodactylus turnbulli* are born with a fully formed daughter in their uterus and this daughter gives birth within 24 h to another fully formed daughter, thus allowing the number of worms on a fish to rapidly increase exponentially (Bakke *et al*., 2007). This parasite can cause mortality due to damage to the epithelial barrier, secondary bacterial infections as well as increased susceptibility to predation (Cable and Van Oosterhout, 2007). The exponential increase of worms on an infected fish, together with the direct transmission, generates epidemic outbreaks in guppy populations whereby the prevalence and worm intensity rapidly increase (Scott, 1982). As individuals die or recover from an infection, the prevalence and worm intensity decrease (Tadiri *et al*., 2019). Once a fish has recovered, it resists re-infection for 4–6 weeks (Scott, 1985) during which the parasite can persist at a low prevalence until enough susceptible hosts are present to allow another epidemic outbreak (Scott and Anderson, 1984). As noted above, a previous study suggests that guppies respond to putative infection cues released from *Gyrodactylus*-infected individuals (Stephenson *et al*., 2018) but the specific changes in behaviour have not been well established (Behringer *et al*., 2018). Additionally, the link between exposure to chemical infection cues and future susceptibility and infection dynamics have not been studied.

### Objectives and hypotheses

The goals of this study were to determine if putative chemical cues released from stimulus guppies infected with *G. turnbulli* affected individual and shoaling behaviour of uninfected male and female test guppies, and whether prior exposure to the putative infection cue would lower the epidemic outbreak profile when the parasite was introduced into a guppy shoal. A secondary goal was to consider whether the sex of the test fish and duration of infection on the stimulus fish influenced the outcomes.

Based on a study showing that overall activity is reduced when guppies are infected (Jog *et al*., 2022), exposure to the chemical infection cue was expected to decrease the overall distance travelled, and the cumulative duration of darting and freezing events. Within a shoal, distance between fish was expected to be greater when the shoal was exposed to the infection cue, as guppies are known to avoid *Gyrodactylus-*infected conspecifics (Croft *et al*., 2011). Based on recent findings by Stephenson *et al*. (2018), it was hypothesized that cues released at the peak of the infection (day 16) would have a stronger impact than cues released earlier (days 4 and 8). As male guppies exhibit bolder behaviour (Piyapong *et al*., 2010) and may be more vulnerable to predation during severe *G. turnbulli* infections due to their smaller size compared to females (Stephenson *et al*., 2016), it was also hypothesized that males would respond more strongly to infection cues than females.

## Methods

### Host and parasite culturing and maintenance

The guppies used in all experiments had been transported from the Turure river shed in Trinidad (GPS coordinates: 10.6903, −61.1638) to Montreal (Quebec, Canda) in 2015, where they were treated with broad-spectrum antibiotics and antifungals, then kept in quarantine for 3 months. After quarantine, they were bred for approximately 20 generations in a laboratory setting free of exposure to any parasitic infections. *Gyrodactylus turnbulli* was isolated from an ornamental guppy purchased from a supplier in Montreal. To ensure the isolated parasites were of the same species and strain, 2 worms from the tail of 1 ornamental guppy were transferred onto a lab-reared guppy that was then maintained in a tank with naïve lab-reared guppies. Over the course of the experiments, this parasite strain was maintained in 2 aquaria to which naïve fish were added weekly (Scott and Anderson, 1984). The parasite species was identified as *G. turnbulli* by measuring 2 features on the opisthaptor: the total length of the hamulus (55.5 ± 0.4 *μ*m) and the width of the ventral bar (29.6 ± 0.5 *μ*m) on 5 parasites from 3 infected fish, based on Harris (1986).

To infect experimental fish, an infected and naïve guppy was anaesthetized in 0.02% tricaine methane-sulphonate (Finquel MS-222, Argent Laboratories, Redmond, WA, USA; MS-222), buffered to a neutral pH with sodium bicarbonate and then placed together in a Petri dish containing the MS-222 solution. Using an SMZ800 stereomicroscope (Nikon, Tokyo, Japan), a scale with a parasite from the donor fish scale was placed on the tail of the recipient fish and observed until the parasite moved onto the recipient fish (Gheorghiu *et al*., 2012).

### Experiment 1: impact of exposure to infection cue on individual guppy behaviour

The goal of this experiment was to determine if exposure to conditioned water from stimulus guppies with mid (D8) to late stage (D16) *G. turnbulli* infection altered the behaviour of individual uninfected test guppies.

The 2 × 2 × 2 experimental design included 2 experimental groups (control or infected stimulus fish), 2 durations of infection on the female stimulus fish (D8 or D16) and 2 sexes of test fish (male or female). A total of 15 test fish of each sex were exposed once only, for 10 min, to the infected stimulus cue either 8 or 16 days after infection of the stimulus fish. A total of 7 test fish of each sex were exposed to a control stimulus cue on D8 or D16. The behaviour of each test fish was recorded by using an overhead video camera during a 10 min trial.

#### Preparation of stimulus cue

Sixteen female Turure guppies were selected from their home tank and temporarily housed in 4 tanks with 4 females each assigned randomly as infection or control stimulus fish. On day 0, 2 females from each tank were assigned as infected stimulus fish, experimentally infected with 2–3 individual *G. turnbulli* worms to ensure at least 1 parasite established on each fish and placed in a separate tank. The 2 females assigned as control stimulus fish were anaesthetized but not infected and also placed in a separate tank.

One day before each trial (day 7 and day 13), the stimulus fish were anaesthetized, their length was recorded and each fish was scanned either to confirm no parasites on the control fish or to count the number of parasites on the infected fish. After recovering from anaesthesia, the 2 infected and the 2 control fish were placed in separate plastic containers with 1 L of water for 24 h without food to obtain ‘conditioned water’ containing the infected or control stimulus cues (Stephenson *et al*., 2018).

#### Experimental room and tanks

**Fig. 1.**
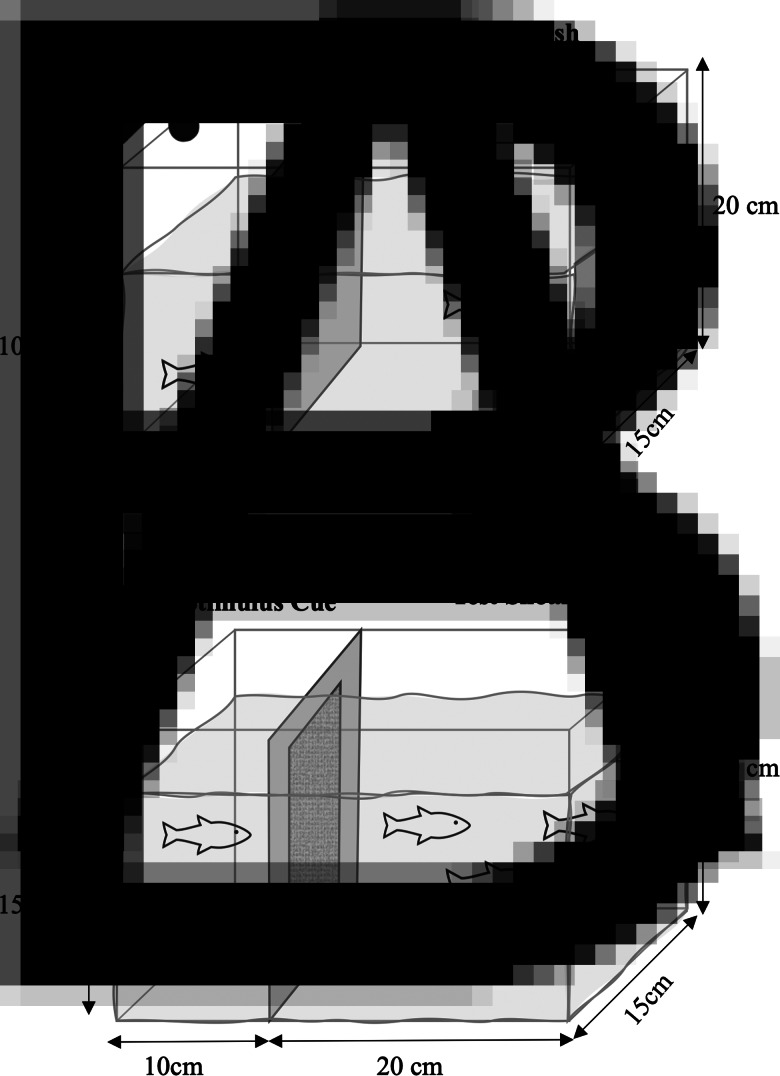
Image of experimental tank (9.4 L). Stimulus and test fish separated by barrier that permitted flow of water and cues but not parasites. (A) Tank for experiment 1. Stimulus cue included both conditioned water and a live female present for the 10 min video trial. (B) Tank for experiment 2 with additional permeable polyester barrier. Two stimulus females lived in the same tank as the 4 test fish for the duration of the experiment. Ten-minute video trials on 0, 4, 8 and 16 days of exposure to stimulus fish. On day 16, stimulus fish were removed, 1 of the test fish was infected and parasite epidemic was monitored every 3 days for 40 days.

The trials took place in a room (3 m × 2 m), artificially lit by standing lamps on 2 side walls and 4 fluorescent ceiling light fixtures near each corner of the room to ensure sufficient lighting to maximize quality of videos. A 9.4 L experimental tank (*L*: 30 cm × *W*: 12.5 cm) was placed in the middle of the room and filled to a depth of 10 cm with dechlorinated water from the lab reservoir. A Plexiglas frame with a plastic screen mesh was inserted to separate the tank into a smaller ‘stimulus’ (10 × 12.5 cm^2^) and a larger ‘test’ (20 × 12.5 cm^2^) compartment (Fig. 1A). An air stone was placed in the stimulus compartment to promote water movement into the test compartment. The outer surface of the stimulus compartment was covered with black cardboard paper to darken it and minimize visual cues observed by the test fish as much as possible.

#### Protocol

On D8 and D16 after experimental infection or sham infection of the stimulus fish, test fish were randomly selected based on treatment, sex and home tank using Windows Excel Rand function, and tested according to the randomized sequence over 4 test days. Between 8 and 12 trials were performed per day, using stimulus fish from a single group. In total, each stimulus fish was used between 4 and 6 times on D8 and D16, however, each trial used a different test fish.

For each trial, an uninfected test fish was placed in the experimental tank test compartment for 10 min acclimation (Croft *et al*., 2006). Then, 100 mL of conditioned water was added to the stimulus compartment and 1 stimulus fish was also added to supplement the stimulus cue. The outer edges and top of the stimulus compartment were then covered with opaque plastic to limit the amount of light in this compartment. The trial was recorded for 10 min. Then, the stimulus fish was returned to its tank and the second stimulus fish was used for the next trial. The test fish was anaesthetized, their length was measured, scanned to confirm that it was uninfected and then placed in a new home tank. The time of day and home tank were recorded. Each test fish was used either on D8 or D16, and each stimulus fish was used for both D8 and D16 trials. The experimental tank was rinsed with 70% ethanol to ensure any residual cue or parasites were removed before the next trial.

### Experiment 2: impact of prior exposure to infection cue on shoal behaviour and parasite population dynamics

The goal was to first study the behavioural response of mixed sex guppy shoals to a putative infection cue over 16 days (shoal behaviour phase) and then to study the impact that prior exposure to the cue for 16 days had on the subsequent outbreak dynamics following introduction of the parasite to the shoal (parasite population dynamics phase).

The shoal behaviour phase used a 2 × 4 repeated design with 2 treatments (exposure to control or infected stimulus fish; 8 shoals per treatment) and 4 periods of continuous exposure to cues (D0, D4, D8 or D16). Each test shoal included 2 female and 2 male uninfected, 4–7-month-old Turure guppies.

#### Experimental tank

The tanks for experiment 1 were adapted for experiment 2 by adding a translucent permeable polyester barrier that prevented the parasites on the stimulus fish from crossing into the larger test compartment (Fig. 1B). The barrier was held in position within a Plexiglas frame. Although no parasites crossed the barrier in experiment 1, this was an added precaution because the infected stimulus fish and test fish were housed in the same tank for much longer than in experiment 1. The behavioural trials took place in the same dark room, with the same lighting as for experiment 1.

#### Phase 1: Protocol for shoal behaviour

Test shoals were acclimated for 4 days in the test compartment along with 2 uninfected adult females in the stimulus compartment. Baseline behaviour of each shoal was then recorded for 10 min (D0). Then, the 2 adult fish from stimulus compartment of each tank were anaesthetized, either infected with 2–3 *G. turnbulli* or used as control stimulus fish, and then returned to the same stimulus compartment. Both control and infected stimulus fish were monitored every 3 days to record the parasite intensity over 16 days, as Stephenson *et al*. (2018) reported that putative chemical *Gyrodactylus* cues released 16 days after infection elicited a behavioural response. Trials for all 16 tanks were conducted on the same day for each exposure period. Tank order was randomized using the Rand function in Microsoft Excel on each day.

On D4, D8 and D16, each experimental tank was transported into the experimental room and the fish were left to acclimate for 15 min (Croft *et al*., 2006). Then, the video recorder was turned on from outside the room and recorded for 10 min. After each trial, all test fish were anaesthetized, their length was measured and they were visually scanned to confirm that they were not infected and returned to their experimental tank.

#### Phase 2: Protocol for parasite population dynamics

To determine whether exposure over 16 days to chemical cues from the infected stimulus fish had an impact on subsequent parasite transmission and population dynamics, the stimulus fish were removed on D16, the length and distinguishing features of each fish in the test shoals were recorded, 1 randomly selected female fish in every test shoal was infected with 3 *G. turnbulli* and returned to the experimental tank. The number of parasites on each test fish in each shoal was recorded every 3 days for 40 days, which ensured that at least 1 epidemic cycle would be monitored.

### Behavioural and infection outcomes

For experiment 1, Ethovision XT 14.0 (Noldus, Wageningen, the Netherlands) animal tracking software was used to quantify behavioural parameters by sampling the recording 25 times per second. For experiment 2, Ethovision XT 11.0 animal tracking software and the social interaction modules were used to record the individual and shoal characteristics at 25 times per second.

#### Individual behaviour activity measures

The *total distance moved* measured the length of the entire track for the 10 min trial. The *latency to first move* was the time before the guppy first exceeded the threshold for detectable movement of 0.75 cm s^−1^. *The duration of ‘darting’ events* was recorded using a multi-condition parameter that determined the cumulative time when the guppy was swimming along the side of the wall at a higher velocity than 2.5 cm s^−1^. This was validated using the integrated visualization feature on the software. The *duration of ‘freezing’ events* was calculated as the cumulative time when velocity was 0 cm s^−1^ for at least 1 s.

#### Individual behaviour location measure

The *duration in the centre of the tank* was calculated based on a software-defined centre zone which equalled half of the total surface area of the experimental tank (125 cm^2^). The duration in the stimulus zone was calculated based on a software-defined rectangle (15 cm × 5 cm) which was the zone of the test fish compartment which was 25% closest to the stimulus source.

#### Shoal behaviour measures

For the shoal behaviour phase of experiment 2, individual behaviour and activity measures were recorded for each fish in the shoal and averaged. In addition, the distance between the centre point of multiple subjects was used as a measure for shoal cohesion, as the software could not distinguish individual fish. The *average inter-fish distance* was measured 5 times each second throughout the trial and averaged for all 4 members of the shoal. The *duration in close proximity* (s) was defined as the average time a subject spent within 0.5 cm of another subject (Green *et al*., 2012). This value was chosen as *G. turnbulli* is known to transfer when an infected fish is within 0.5 cm of another fish (Harris and Lyles, 1992).

For both individual and shoal outcomes, the threshold set for subject loss due to misdetection by video-tracking software was <5%. If the subject loss exceeded 5% for an individual trial, the tracks were manually edited to add tracks for the portions where the fish had not been detected (Green *et al*., 2012).

#### Epidemic outcomes

For the parasite population dynamics phase of experiment 2, the *prevalence* (per cent of infected fish in each shoal) was averaged across shoals at each time point. The *intensity* (number of parasites) on each fish in the shoal was recorded and averaged on a given day for each shoal.

### Statistical analyses

The raw data were exported from the Ethovision XT software into an excel file which was uploaded to R statistical software 4.1.1 (Team, 2013). Boxplots or scatter plots (ggplot2 package) were created for each variable to provide visual representations of the data (Wickham, 2011). Power analyses were completed for all variables using the function pwr.f2.test (pwr package) (Champely *et al*., 2017). Unless otherwise stated, all values are mean ± s.e.m.

For experiment 1, linear models or generalized linear models (GLMs) were used to analyse behavioural variables using lm and glm functions (lme4 package), respectively (Bates *et al*., 2007). A gamma distribution was used for all behavioural variables except for the *duration of freezing events* which was normally distributed (Team, 2013). In all models, the treatment, duration of infection on stimulus fish (D8 or D16) and sex of test fish were included as fixed effects. The best fit for each model was selected based the lowest Akaike information criterion (AIC). The time of day when the trial was recorded, and interactions were included in a model if they significantly improved the model AIC. The length of the test fish, length of the stimulus fish and number of worms on the stimulus fish were also included in all initial models. For the *duration in the stimulus zone*, only the length of the test fish improved the model and was significantly associated with the response variable, thus was retained. For all other models, the 3 variables were excluded from the final analysis as they did not improve model AIC and were not significantly associated with response variables.

As phase 1 of experiment 2 provided repeated measures of individual and shoaling behaviour, linear mixed models (LMMs) or generalized linear mixed models (GLMMs) were used to assess behavioural outcomes depending on distribution of the data. In all models of individual and shoal behaviour in phase 1, both treatment and duration of cue exposure (D0, D4, D8, D16) were included as fixed effects, and tank was included as a random intercept in the model using lmer and glmer (Bates *et al*., 2007). The time of day when the trial was recorded was included as a fixed effect if it significantly improved the model AIC. The number of worms on the stimulus fish, and lengths of the test fish and stimulus fish were excluded in the final analysis as they did not significantly improve the model AIC and were not significantly associated with the response variables. All models used normal distributions except for *total distance moved* and *latency to first move* which followed gamma distributions.

Parasite population dynamics (experiment 2, phase 2) were analysed using LMM (*prevalence*) with log-transformed data using the lmer function (lme4 package) and GLMM using a negative binomial distribution (*intensity*) using the glmer.nb function (lme4 package). Both *prevalence* and *intensity* models included tank, the stimulus cue, length of test fish and days post-infection as fixed effects, as well as days post-infection squared given that the relationships between days post-infection and both *prevalence* and *intensity* followed a polynomial curve. The *intensity* model also included the random effect of fish as individuals were repeatedly sampled. Then, values which predicted worm intensity for each day post-infection were extracted from the model using the augment function of the broom.mixed package (Ben Bolker, 2022). As models used a negative binomial distribution with a log-link function, predicted worm intensities were back transformed by raising the log-predicted number to exponent 10, then plotted for each stimulus cue group over time (ggplot2 package) with confidence intervals from the geom_smooth function (Wickham, 2011).

For both experiments, significance (*P* < 0.05) was tested using the summary function (base R package) and post-hoc pairwise comparison tests were performed using the emmeans function (emmeans package) (Lenth *et al*., 2018). Normality, independence and homogeneity of variances were assessed to ensure the data met model assumptions (Bolker *et al*., 2009).

## Results

### Experiment 1: exposure to an infection cue impacts behaviour of individual guppies

The stimulus fish used to prepare the control conditioned water were larger (27.5 ± 0.7 mm) than the infected stimulus fish (25.0 ± 0.5 mm) (*t*_56.9_ = −2.7, *P* = 0.009), but the length of the stimulus fish did not impact test fish behavioural or location outcomes. The number of parasites on the infected stimulus fish was higher on D16 (125.9 ± 7.4) than on D8 (44.2 ± 1.8) (*t*_32.6_ = −10.6, *P* = 3.5 × 10^−12^). The lengths of the test fish exposed to the infection cue or the control cue on D8 or D16 did not differ (22.0 ± 0.4 mm).

#### Activity outcomes

**Fig. 2.**
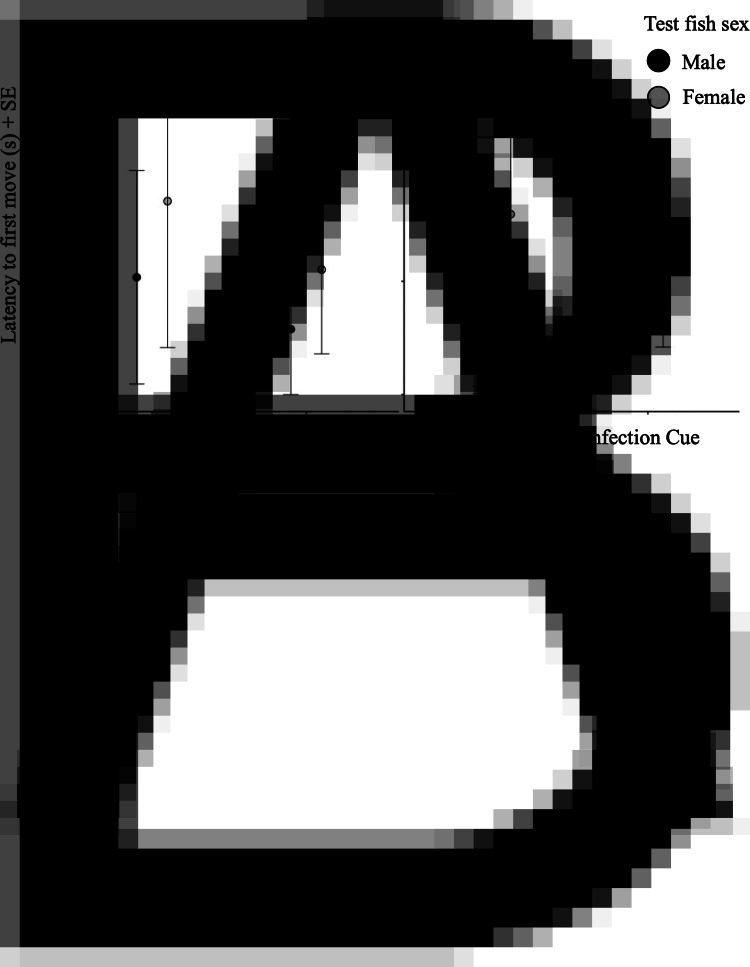
Least-squares means ± s.e. for latency to first move when an individual guppy was exposed for 10 min to water conditioned with chemical infection cues released from guppies that had been infected with *Gyrodactylus turnbulli* for 8 days (A) or 16 days (B). The significant interaction between treatment and sex on day 16 is represented by lower case letters.

Total distance moved, duration darting and duration freezing were not influenced by our experimental treatment during the 10-min trial (Table 1), but all 3 differed by sex of the test fish and day of the trial. Males moved more, ‘darted’ more and ‘froze’ less than females (Table 1). On D16, both male and female guppies moved more, ‘darted’ more and ‘froze’ less than on D8 (Table 1). Significant treatment × sex and treatment × sex × day interactions were detected for latency to first move (Table 1, Fig. 2). On D16, but not on D8, males exposed to the control cue had a very low latency to begin moving, resulting in a significant outcome between males on day 16.

**Table 1.** Impact of exposure to *Gyrodactylus turnbulli* infection cue or control cue on behaviour of individual uninfected guppies

Cue	Sex	Total distance (cm)	Duration darting (%)	Duration freezing (%)	Latency to first move (s)	Duration in centre of tank (%)	Duration in stimulus zone (%)
Infection	Male	508.1 ± 90.8 (343.6)	12.5 ± 3.2 (4.7)	60.5 ± 5.7 (73.6)	48.5 ± 11.4 (20.1)	24.4 ± 4.4 (23.0)	51.73 ± 6.0 (40.1)
Control	Male	661.9 ± 172.5 (459.4)	18.1 ± 5.8 (9.5)	51.6 ± 9.9 (58.8)	44.0 ± 36.6 (2.7)	15.9 ± 4.8 (12.8)	46.8 ± 9.1 (29.5)
Infection	Female	347.2 ± 88.8 (299.6)	6.7 ± 2.0 (1.8)	70.9 ± 4.7 (73.6)	65.7 ± 19.1 (27.9)	36.0 ± 6.1 (22.1)	48.4 ± 6.0 (39.0)
Control	Female	369.7 ± 88.8 (252.7)	8.7 ± 2.9 (4.4)	68.5 ± 7.0 (77.7)	67.8 ± 21.2 (18.4)	22.3 ± 4.2 (23.9)	54.7 ± 7.4 (55.4)
GLM results							
Treatment		−0.13 ± 0.20	−0.31 ± 0.26	4.9 ± 6.5	−0.7 ± 0.6	0.44 ± 0.21*	−0.06 ± 0.48
Sex: male		0.41 ± 0.19*	0.56 ± 0.24*	−12.9 ± 6.1*	−3.1 ± 0.7***	−0.37 ± 0.19	0.01 ± 0.13
Day post exposure		−0.57 ± 0.19**	−0.81 ± 0.24**	15.5 ± 6.1*	0.5 ± 0.7	−0.09 ± 0.19	0.21 ± 0.13
Time of day		−0.46 ± 0.21*	−0.72 ± 0.26**	NI	1.1 ± 0.3**	NI	NI
Treatment × sex		NI	NI	NI	3.3 ± 0.8***	NI	NI
Treatment × sex × day		NI	NI	NI	−2.9 ± 1.3*	NI	NI

Pooled data from fish exposed to mid (D8) and peak (D16) stages of infection cues. Mean ± s.e. (median) for each outcome and GLM output model estimates ± s.e. Variables not included in the model are represented by NI (not included).

Significance of model estimates: *P < 0.05; **P < 0.01; *** *P* < 0.001.

#### Location outcomes

Time spent in the centre of the tank was significantly affected by treatment but not by sex or day of the trial (Table 1). Guppies exposed to the infection cue spent more time in the centre of the tank whereas guppies exposed to the control cue spent more time in the periphery of the tank on both D8 and D16. However, guppies exposed to the infection cues did not avoid the stimulus compartment as indicated by a similar proportion of time in the zone of the test arena which was closest to the stimulus compartment (Table 1).

### Experiment 2: exposure to an infection cue does not alter shoaling behaviour but prior exposure to an infection cue delayed and dampened increase in parasite intensity in guppy shoal

Test fish exposed to the infection (16 males and 16 females) and the control (16 males and 16 females) cue were of similar length (16.8 ± 0.3 mm), as were control (16 females) and infected (16 females) stimulus fish (19.4 ± 0.7 mm). The number of parasites on the infected stimulus fish increased over time (D4: 19.1 ± 2.7; D8: 31.1 ± 7.2; D16: 49.42 ± 19.9; *F*_(3, 28)_ = 3.75, *P* = 0.022).

#### Phase 1: activity, location and social interactions during ongoing exposure to infection cues

Exposure to infection cues did not alter how far shoals moved, duration of ‘freezing’ events, latency to first move or the proportion of time spent in the centre of the tank. However, as the trials progressed from D4 to D16, all shoals began to move more, freeze less and fish spent more time in closer contact (Table 2).

**Table 2.** Impact of prolonged exposure to *G. turnbulli* infection cue or control cue on uninfected guppy shoal behaviour

	Total distance moved (cm)	Duration of ‘freezing’ events (%)	Latency to first move (s)	Duration in centre of tank (%)	Average inter-fish distance (cm)	Duration in close proximity (%)
Treatment	−0.06 ± 0.11	−2.3 ± 3.8	0.02 ± 0.25	−0.06 ± 0.14	−0.004 ± 0.32	0.4 ± 1.3
Time of day	NI	NI	0.68 ± 0.25**	0.39 ± 0.14**	NI	NI
Duration of prior exposure						
4 days	−0.20 ± 0.07***	10.7 ± 2.8***	−0.06 ± 0.27	0.34 ± 0.16*	0.44 ± 0.37	−3.8 ± 1.6*
8 days	0.13 ± 0.07	−7.1 ± 2.8*	−0.51 ± 0.23*	−0.52 ± 0.14***	−0.85 ± 0.37*	5.1 ± 1.6**
16 days	0.09 ± 0.07	−5.7 ± 2.8	−0.013 ± 0.23	−0.44 ± 0.14**	−1.2 ± 0.37**	7.3 ± 1.6***

GLMM output estimates (±s.e.) of fixed effects on shoal behaviour when groups of 4 guppies were continuously exposed to cues from 2 *G. turnbulli*-infected guppies over 16 days. Tank used as random intercept. Variables not included in model are represented by NI (not included).

Significance of model estimates: *P < 0.05; ** *P* < 0.01; *** *P* < 0.001.

**Fig. 3.**
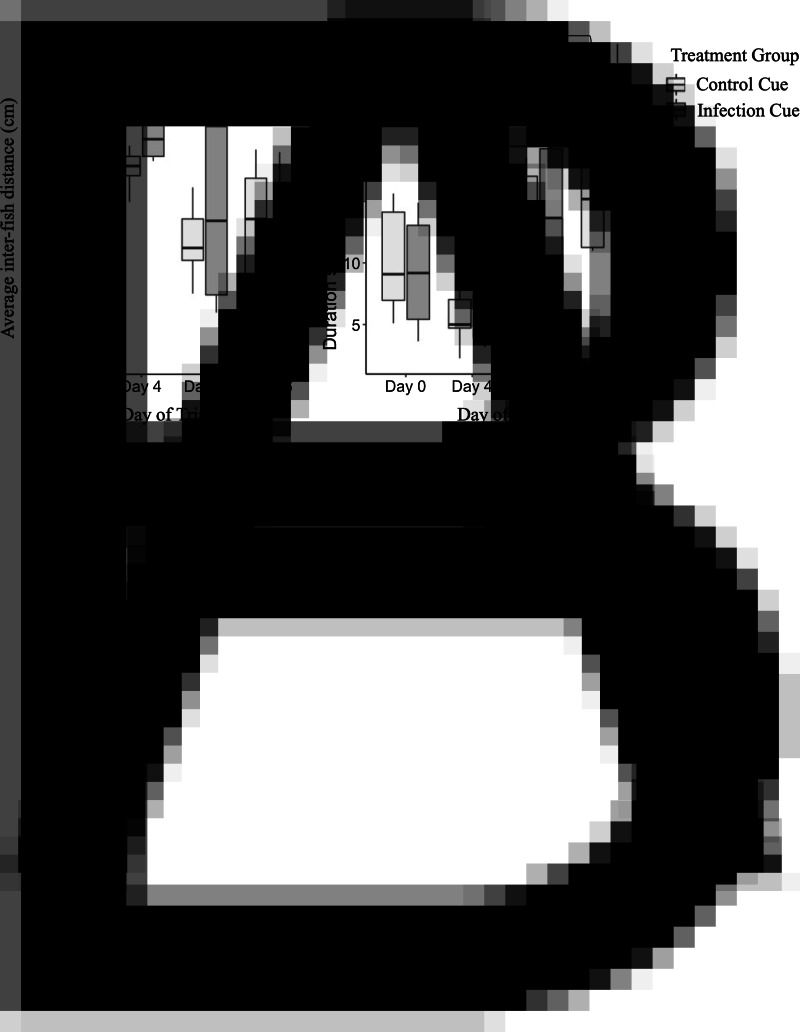
Boxplots of social interaction variables for experiment 2 (phase 1). Average inter-fish distance (A) and average duration within 0.5 cm of another fish (B) for shoals of guppies when exposed to continuous chemical cues from *G. turnbulli-*infected conspecifics for 0, 4, 8 or 16 days. Comparison bars represent significant effects of day of trial for pooled control and treatment groups. *P < 0.05, **P < 0.01, ***P < 0.001.

Neither the inter-fish distance nor the duration spent in close proximity was impacted by exposure to continuous infection cues at any time during the trials (Fig. 3). However, both social interaction outcomes responded to duration of exposure to the cue. The average inter-fish distance was shorter on D8 and D16 compared to D0 (Fig. 3A) and the duration of time in close proximity was lower on D4 but higher on D8 and D16 compared to D0 (Fig. 3B).

#### Phase 2: parasite population dynamics

Infection of 1 fish within the shoal established an epidemic in 7 shoals that had been exposed for 16 days to the infection cue and 6 shoals that had been exposed for 16 days to the infection cue. Throughout the 40 days of experiment 2, phase 2, 5 infected guppies exposed to cues from infected fish and 4 infected guppies exposed to cues from control fish died.

**Fig. 4.**
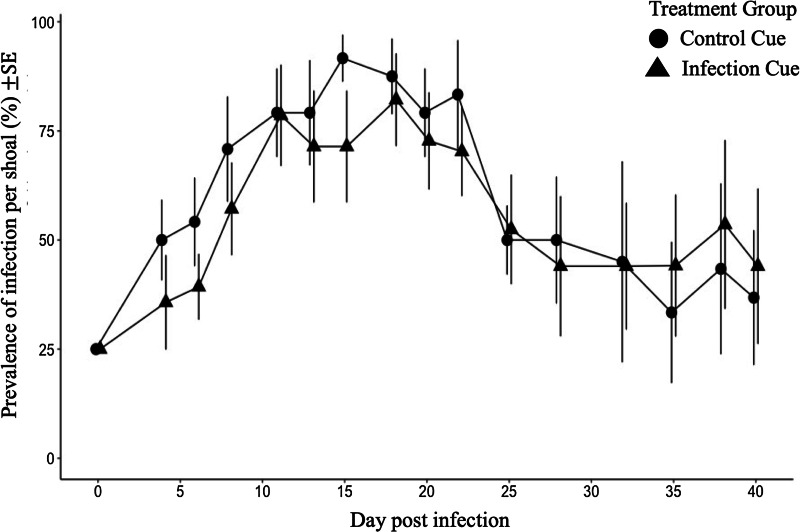
Prevalence (%) of *G. turnbulli* in guppy shoals over 40 days post-infection (data points for guppies exposed to infection cue are positioned slightly to the right to avoid overlap of s.e. bars). Shoals had been previously exposed to chemical cues from *G. turnbulli*-infected fish or control fish for 16 days. LMM estimates based on natural log of proportion of fish infected per shoal, centred around the mid-point (day 20): infection cue = 0.04, NS; day post-infection = 0.001, NS; infection × day post-infection = 0.001, NS.

**Fig. 5.**
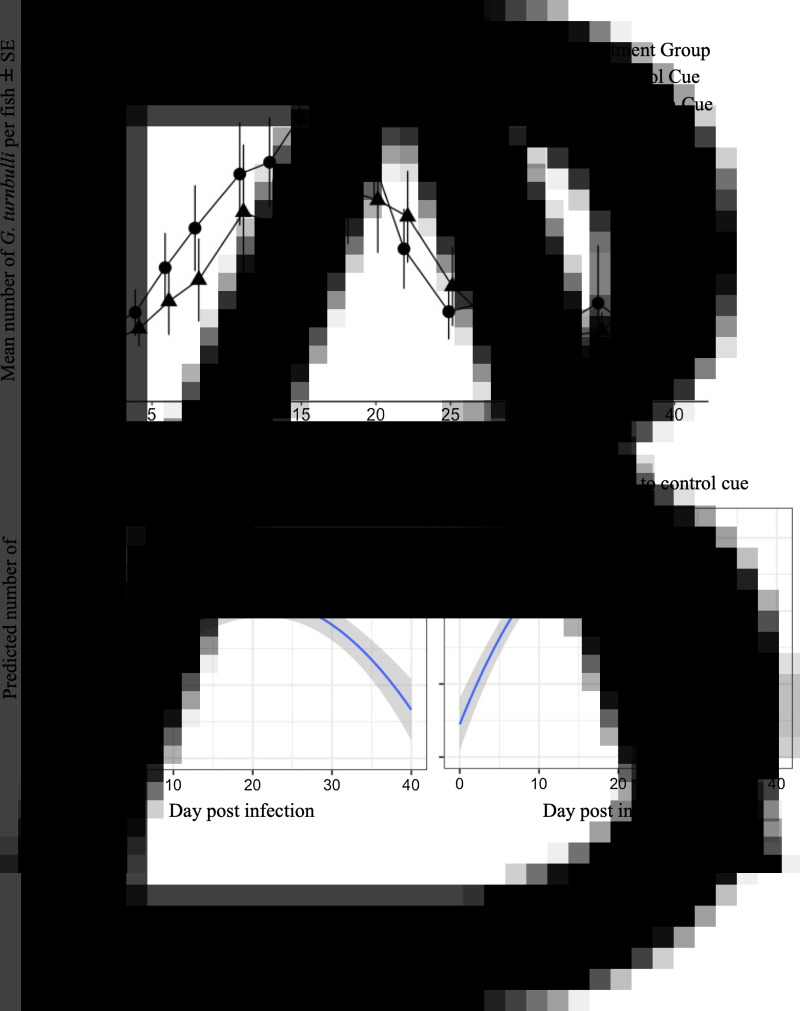
Intensity of *G. turnbulli* on guppies over 40 days post-infection. (A) Mean intensity over time for guppies that had been exposed to chemical cues from *G. turnbulli*-infected fish or control fish for 16 days prior to introduction of the parasite into the shoal (data points for guppies exposed to infection cue are positioned slightly to the right to avoid s.e. bar overlap), centred around the mid-point (day 20): infection cue = −0.6, NS; sex (male): −0.12, NS; day post-infection = −0.01, NS; infection × day post-infection = 0.03, *P* = 0.008. GLMM (negative binomial distribution) predicted values for parasite intensity during outbreak of *G. turnbulli* infection in guppies that had been exposed for 16 days to chemical cues from *G. turnbulli*-infected fish (B) or cues from control fish (C). Black dots represent peak day post-infection.

The *prevalence* (Fig. 4) and *intensity* (Fig. 5A) of infection increased and then decreased over 40 days regardless of whether shoals had previous exposure to an infection cue. Prior exposure to the infection cue did not impact the prevalence of guppies infected over time. However, exposure to the infection cue dampened and delayed the peak intensity as shown by the GLMM for intensity (Fig. 5A; *P* = 0.008). Shoals that had been exposed to the infection cue had a predicted lower and delayed peak intensity (4.6 worms and 22 days, respectively; Fig. 5B) compared with those exposed to the control cue (6.4 worms and 18 days, respectively; Fig. 5C). The sex of the guppy did not impact worm intensity.

## Discussion

The concept that infected hosts release chemical cues that warn conspecifics of the threat of infection has only been studied in a few aquatic systems (Kiesecker *et al*., 1999; Poulin *et al*., 1999; Stephenson *et al*., 2018). Using the guppy skin ectoparasite, *G. turnbulli*, behavioural data on individual test fish showed that guppies responded and were presumably able to detect putative infection cues as guppies exposed to this cue spent more time in the centre of the tank, and the initial movement of males was delayed in response to the stimulus cue released 16 days after infection. Although shoaling behaviour was unaffected by exposure to the infection cue, prior prolonged exposure to the infection cue dampened and delayed the peak intensity of infection following introduction of *G. turnbulli* into the guppy shoals, perhaps because the putative infection cue induced a degree of protection. Together, these findings provide additional evidence that *Gyrodactylus-*infected guppies release a chemical cue that has subtle but detectable effects on behaviour of isolated, uninfected guppies and show for the first time that exposure to this cue dampens the epidemic profile once shoals are exposed to direct infection.

Prior evidence that chemical cues from parasitized fish affect the behaviour of an individual uninfected host is limited. Chemical cues released during movement of cercariae through the epidermis and dermis into the muscle tissue of rainbow trout have been shown to induce increased darting but most of the time remained motionless in conspecifics (Poulin *et al*., 1999). In the present study, neither darting nor initiation of movement was affected by exposure of guppies to the putative *G. turnbulli* chemical cue. Difference in behavioural responses to cues released during cercarial penetration and *G. turnbulli* infection may, in part, be due to differences in the host–parasite interaction in the skin. In contrast to cercariae, *G. turnbulli* use hooks to attach to, and move over the skin of guppies. These hooks continuously pierce the upper layers of the guppy epidermis (Bakke *et al*., 2007; Gheorghiu *et al*., 2012) but do not directly damage the underlying dermis or muscle. The interaction with cercariae is brief as it takes only a few minutes for cercariae to penetrate the host epithelium (Poulin *et al*., 1999) and fish often swim through a swarm of cercariae (Poulin and FitzGerald, 1989). Thus, in response to cues of cercarial infection, rapid darting then pausing may be an effective way to avoid swarms of cercariae. In contrast, the risk for *G. turnbulli* infection is close contact with an infected fish and fish can remain infected for a few weeks (Gheorghiu *et al*., 2012). Fish that detect an infection cue may more easily escape any potential direct fish-to-fish transmission of *G. turnbulli* if they are in an area that allows for movement in any direction. This is consistent with the present finding that test fish exposed to infection cues spent more time in the centre than the periphery of the experimental tank.

Stephenson *et al*. (2018) reported that uninfected conspecifics avoided the area where a *G. turnbulli* cue was being released but only when cues were collected at 16 days after *G. turnbulli* infection. In the present study, the shift to the centre of the tank was detected at both 8 and 16 days after infection of the stimulus fish. This could be explained by slight differences in preparation of the chemical stimulus. Stephenson *et al*. (2018) used frozen conditioned water for the chemical stimulus whereas the putative cue in this study was a combination of fresh conditioned water and a live stimulus fish placed in the stimulus compartment of the tank. Also, experimental tanks in the present study were smaller. Together, these differences may have resulted in a stronger concentration of stimulus cue on day 8. A longer lag before males began responding to the D16 infection stimulus cue compared with the control cue was also detected. We suspect that this result may be due to the unexpectedly low mean and extremely low standard error for latency to first move in the fish exposed to control cue, rather than a longer lag in fish exposed to infected cues.

Shoals of guppies respond to the introduction of a *G. turnbulli-*infected fish (Croft *et al*., 2011; Reynolds *et al*., 2019) but the present study is the first to explore the impact of an infection cue on shoaling. When a *G. turnbulli*-infected individual was introduced to a shoal of guppies in a stagnant pool, the distance between members was greater and frequency of contacts between shoal members of the population was reduced when compared to shoals where a healthy individual was introduced (Croft *et al*., 2011). In an experimental system which exposed guppies to different water-flow regimes and different rates of parasite abundance, the distance between shoal members increased as prevalence and intensity of parasites increased in the zero-flow regime, but this difference was not observed in high water-flow regimes (Reynolds *et al*., 2019). The authors suggested a trade-off between the potential benefit of reducing infection risk by avoiding shoal mates and the benefit of energy conservation *via* shoaling. This would be especially relevant in high-flow systems where conserving energy to swim against the current may be more important than avoiding infection (Reynolds *et al*., 2019). In contrast to these findings, none of the shoaling behavioural outcomes was affected by exposure to the infection cue in the present study. This could be explained by different protocols. Although Croft *et al*. (2011) and Reynolds *et al*. (2019) observed behavioural differences in the shoal network when they introduced infected individual fish that were able to physically touch and interact with the shoal, the present study used chemical cues from infected individuals.

On comparing behavioural results between experiment 1 and experiment 2, phase 1, it is interesting to note that the higher time spent in the centre of the tank in response to short-term exposure to the infection cue was not observed in experiment 2 where guppy shoals were exposed to a continuous cue. It is possible that guppy–guppy interactions (Jog *et al*., 2022) may have increased guppy use of the entire tank. However, as we were not able to track movement of individual guppies in the shoal, we aren't able to explore this possibility. Additionally, as guppies use both visual cues and chemical cues to detect risk in their environment (Stephenson, 2016), it is possible that guppies in these shoals could recognize that their immediate shoal mates posed no infection risk, thus did not alter their behaviour.

The social network of all shoals gradually became more clustered over 16 days as evidenced by increased movement of the shoal, less freezing and shorter inter-fish distance over time, indicating habituation to trial procedures or acclimation to their shoal mates. As fish preferentially shoal with familiar conspecifics (Krause *et al*., 2000), guppies may have become acclimated to their shoal mates and preferentially shoaled in a tighter network as time spent together in the same tank increased.

*Gyrodactylus* spp. are responsible for major outbreaks in aquaria (Schelkle *et al*., 2009), aquaculture (Masud *et al*., 2019) and the wild (Denholm *et al*., 2016) where they can cause considerable mortality. The epidemic dynamics of *G. turnbulli* have been explored by tracking changes in prevalence and parasite intensity in experimental guppy populations (Scott and Anderson, 1984; Richards and Chubb, 1996; Tadiri *et al*., 2013, 2019), in semi-natural mesocosms (Pérez-Jvostov *et al*., 2012), and in theoretical studies (Scott and Anderson, 1984; Van Oosterhout *et al*., 2008). Typical of other microparasite epidemics, the prevalence profile increases as the infection first spreads through the population and then declines as infected hosts become resistant and lose their parasites or die. *Gyrodactylus turnbulli* have been shown to be more likely to leave a more heavily infected host (Stephenson *et al*., 2017) and less likely to move onto a previously infected host (Scott and Robinson, 1984; Scott, 1985). Fortunately, with the *G. turnbulli*–guppy system it is possible to track the temporal pattern of infection intensity on individual fish because *G. turnbulli* are visible on the surface of the guppies. This provides more detailed insights into processes that influence an epidemic outbreak as, unlike prevalence, changes in parasite intensity in a population are driven primarily by parasite reproduction and survival because movement of parasites among hosts does not affect the average intensity in the host population.

In the second phase of experiment 2, the prevalence profile was unaffected by whether shoals had previously been exposed for 16 days to the infection cue. This observation is consistent with the absence of detectable impacts on shoaling behaviour in the first phase of experiment 2, as fish–fish contact is the primary method of transmission (Harris and Lyles, 1992). Guppies may have imprinted on the infection cue, as demonstrated by Stephenson and Reynolds (2016), which would potentially mean that, once infected, these guppies avoided each other less than shoals exposed to control cues (Stephenson and Reynolds, 2016). However, the prevalence of infection was not different between exposure groups thus if imprinting did occur, it did not result in a more rapid spread of infection. In contrast, the intensity profile was affected by prior exposure to the infection cue, in a direction that suggested the exposure to the cue reduced the growth rate of the parasite population. The serial polyembryony and short generation time characteristic of *G. turnbulli* (Van Oosterhout *et al*., 2007) allow the rapid initial expansion of the parasite population. The first daughter is typically born after 1 day, subsequent births occur at intervals of 2 or 2.5 days, and the average life span is 4.2 days (Scott, 1982). As shown by the predicted values from the GLMM using a negative binomial distribution, the parasite intensity increased more slowly and to a lower peak when guppies had prior exposure to chemical infection cue. Following a second ‘challenge’ infection, parasite numbers increase more slowly and recovery is more rapid (Scott, 1985), indicating development of partial resistance and consistent with the observed intensity profile in response to the chemical cue. Thus, despite no impact of the infection cue on fish–fish transmission, the slower accumulation of parasites suggests that parasites on guppies with prior exposure to the infection cue had a lower rate of parasite reproduction and/or a higher death rate.

In response to a first *G. turnbulli* infection, the epidermis becomes thicker and mucous is released onto the skin surface resulting in a decrease in number and size of mucus cells (Gheorghiu *et al*., 2012), a response that contributes to recovery from infection (Bakke *et al*., 2007). Therefore, it is conceivable that exposure to the chemical cue may have induced such an epithelial immune response in the test fish that provided partial resistance when the test fish was exposed to the actual infection. This interpretation is consistent with the literature suggesting chemical cues released from injured fish that elicit an immune response (Meuthen *et al*., 2020), and that specialized cells called club cells, which are related to chemical communication, may also be involved in the innate immune response for teleost fishes (Alesci *et al*., 2022). If confirmed in further studies using larger host populations living in a more natural setting, this could have important implications for controlling *Gyrodactylus* epidemics especially in the aquarium industry and aquaculture. However, it would require determining the nature of the chemical infection cue.

Although the aim was not to determine the nature of the chemical cue, these results provide some insights. (1) The chemical nature of the *G. turnbulli* cue appears to be distinct from predator alarm cues. Even at low concentrations, predator alarm cues alter the behaviour of fish mainly by increasing the occurrence of freezing and darting (Brown, 2003; Stephenson, 2016). Similar behavioural responses have been detected in response to cercarial penetration (Poulin *et al*., 1999), and hence much of the early literature assumed that the composition of infection cues would be similar to that of predator alarm cues (Kiesecker *et al*., 1999; Poulin *et al*., 1999). However, a different behavioural response was observed in response to *G. turnbulli* cues, perhaps because this ectoparasite remains on the surface of the host, attached to the scales. Additionally, this putative chemical infection cue may have a more subtle impact on behaviour than predator alarm cues, especially if the main benefit of a response to the infection cue is development of partial resistance rather than evading predation. (2) The source of the cue appears not to be from the parasite itself, as there was no relationship between the number of parasites on the stimulus fish and behavioural or epidemic responses of the test fish. These results contrast predator alarm cues where the behavioural response is dependent on the concentration of the cue (Mirza *et al*., 2006). Therefore, the behavioural and epidemiologic effects induced in response to the infection cue are more likely due to physiological or immunological changes in the test fish, such as changes in the guppy epithelium (Gheorghiu *et al*., 2012), expression of immune-related genes (Konczal *et al*., 2020) or increased cortisol levels (Stoltze and Buchmann, 2001; Reynolds *et al*., 2018) than to the parasite load on the stimulus fish. The putative infection cue could also be associated with the skin microbiome of infected stimulus fish. Ectoparasites have been shown to alter the abundance and species diversity of the fish skin microbiome (Vasemägi *et al*., 2017; Kashinskaya *et al*., 2021), and the fish microbiome has been shown to mediate fish behaviour through metabolic processes (Soares *et al*., 2019) which can influence chemical communication between fish conspecifics (Ezenwa and Williams, 2014). Taken together, these insights suggest that further studies on the nature and source of the putative chemical infection cue may more profitably focus on host by-products released during infection, and the skin microbiome than on parallels with predator alarm cues.

The present study had a few limitations. (1) Although sample sizes were small, we were able to detect significant differences. For non-significant variables, power analyses indicated that larger sample size would not have revealed a difference. (2) Guppies had been reared in the lab for many years in the absence of parasites (Blondel, 2021) and the parasite was isolated from an ornamental guppy. Thus, both the observed behavioural responses to infection cues may have been lower and the delayed and dampened parasite intensity profile may have been more pronounced than they would have been had we tested wild guppies that would have had ongoing exposure to a natural *G. turnbulli* infection. Any consequence of repeated exposure to infection cues would need to be evaluated. (3) Every effort was made to keep the minimize light in the stimulus compartment to minimize potential visual cues from the stimulus fish. Despite this, it is possible that the test fish detected visual cues from the stimulus fish. (4) The computer software used had many advantages such as calculating precise tracks of the fish moving in a tank. Previous studies have manually tracked freezing as events staying immobile of 30 s of more (Brown, 2003), we were unable to program the software to distinguish fish that remained still for the full 30 s interval from fish with more than 1 short freezing bout within a 30 s interval. Therefore, our best indicator of freezing was the cumulative duration. The same challenge applied to the frequency of darting as well. (5) For phase 1 of experiment 2, the tracking software often switched the animal identities when they were in close contact with a shoal mate. Therefore, it was only possible to report behavioural responses as the average of the 4 shoal members. Additionally, we were unable to test if guppy shoals spent more time in the stimulus zone as we no longer had access to the version of the software which allows for tracking multiple animals during manuscript revisions. (6) The concentration of the putative chemical cue was not measured and may have differed among trials.

In conclusion, for the first time, evidence is provided that prior exposure to chemical cues released from *G. turnbulli*-infected guppies dampened and delayed the peak worm burden during an epidemic outbreak. Further support was provided that guppies respond behaviourally to chemical cues released by infected fish. Although the mechanism linking the behavioural differences and altered epidemic dynamics are unclear, new observations and questions were generated about the source of this putative infection cue and how it may impact guppy–*Gyrodactylus* ecology.

## Data Availability

The data that support the findings of this study are available from the corresponding author (M. E. S.), upon reasonable request.
